# Multi-Robot Interfaces and Operator Situational Awareness: Study of the Impact of Immersion and Prediction

**DOI:** 10.3390/s17081720

**Published:** 2017-07-27

**Authors:** Juan Jesús Roldán, Elena Peña-Tapia, Andrés Martín-Barrio, Miguel A. Olivares-Méndez, Jaime Del Cerro, Antonio Barrientos

**Affiliations:** 1 Centre for Automation and Robotics (UPM-CSIC), Universidad Politécnica de Madrid, José Gutiérrez Abascal, 2, 28006 Madrid, Spain; elena.ptapia@alumnos.upm.es (E.P.-T.); andres.mb@upm.es (A.M.-B); j.cerro@upm.es (J.D.C.); antonio.barrientos@upm.es (A.B.); 2 Interdisciplinary Centre for Security, Reliability and Trust (SnT), University of Luxembourg, Richard Coudenhove-Kalergi, 6, L-1359 Luxembourg, Luxembourg; miguel.olivaresmendez@uni.lu

**Keywords:** multi-robot, operator interface, situational awareness, immersion, prediction, virtual reality, machine learning

## Abstract

Multi-robot missions are a challenge for operators in terms of workload and situational awareness. These operators have to receive data from the robots, extract information, understand the situation properly, make decisions, generate the adequate commands, and send them to the robots. The consequences of excessive workload and lack of awareness can vary from inefficiencies to accidents. This work focuses on the study of future operator interfaces of multi-robot systems, taking into account relevant issues such as multimodal interactions, immersive devices, predictive capabilities and adaptive displays. Specifically, four interfaces have been designed and developed: a conventional, a predictive conventional, a virtual reality and a predictive virtual reality interface. The four interfaces have been validated by the performance of twenty-four operators that supervised eight multi-robot missions of fire surveillance and extinguishing. The results of the workload and situational awareness tests show that virtual reality improves the situational awareness without increasing the workload of operators, whereas the effects of predictive components are not significant and depend on their implementation.

## 1. Introduction

Multi-robot missions have experienced a noticeable growth over the last decades. Their performance has been improved significantly, and their range of application has been extended. Currently, these missions are applied in multiple domains (air, ground and sea) for diverse purposes (surveillance, search and rescue, environmental monitoring...).

The control and monitoring of these kind of missions causes a series of problems related to human factors. Table 1 collects some of these issues, as well as the consequences for the mission, the procedures to detect them, and the actions to compensate them. According to [1], the most relevant problems in scenarios with multiple robots and single operator are peaks of workload and lack of situational awareness. According to [2], the use of robots in urban search and rescue scenarios has a bottleneck in situational awareness, which can be addressed by considering the robots as sources of information and fusing this information properly.

Workload can be defined as the sum of the amount of work, the working time and the subjective experience of the operator [3]. However, the study of workload usually takes into account multiple attributes (input load, operator effort and work performance) [4] and dimensions (physical and mental demand) [5].

The operators of multi-robot missions have to perceive information, understand the situation, make decisions and generate commands. In this context, excessive workload can lead to an increase of waiting times, errors in decision making and, therefore, a decrease in mission performance [6].

The most common method to determine the workload of a mission is the NASA Task Load Index (NASA-TLX) [5]. This method uses a questionnaire where the operators can perform a subjective evaluation of their workload by answering a set of questions. In the first part of the questionnaire, the operators compare some variables according to their influence on workload: mental demand (low–high), physical demand (low–high), temporal demand (low–high), performance (good–poor), effort (low–high) and frustration (low–high). In the second one, they rate these variables regarding their experience during the mission. The results of 20 years of experiments with NASA-TLX questionnaire have been summarized in a more recent publication [7].

Situational awareness can be defined as the perception of elements in the environment within a volume of time and space (level 1), the comprehension of their meaning (level 2), and the projection of their status in the near future (level 3) [8].

The operators of multi-robot missions have to know not only the location and state of the robots in the scenarios, but also their meaning in the mission and their potential evolution in the near future. In fact, the consequences of a lack of situational awareness are diverse: from operator mistakes to robot accidents.

The most common method to estimate situational awareness is the Situational Awareness Global Assessment Technique (SAGAT) [9]. This method provides an objective measurement and works in the following manner:

1. The operator is watching a simulation of a multi-robot mission.
2. At a certain time of the mission, the simulation is stopped and the interface is blanked.
3. The operator is asked a series of questions about the situation.
4. After the end of the mission, the real and perceived situations are compared.
5. A score is determined in three zones (immediate, intermediate and long-range).

As shown in Table 1, some of the potential solutions for human factor problems in multi-robot missions are related to operator interfaces. Some examples are: the reduction of workload, by adjusting the level of autonomy of the system, or transferring functions from operator to interface; and the improvement of situational awareness, by taking advantage of immersion, or selecting the most relevant information at all times.

This paper analyzes the best-suited operator interfaces for multi-robot systems. On one hand, the main interface design resources have been studied: multimodal interactions (transmission of information through audio and vibration), immersive devices (use of commercial virtual reality headsets), predictive capabilities (application of neural networks to discover information) and adaptive displays (selection of the most relevant information). On the other hand, four interfaces have been developed: a conventional (CI), a predictive conventional (PCI), a virtual reality (VRI) and a predictive virtual reality (PVRI) interface. Finally, the four interfaces have been used by a set of operators to monitor a series of multi-robot missions, and their performance has been evaluated by means of workload and situational awareness tests.

The rest of the paper is organized as follows: Section 2 collects the state of the art about operator interfaces for robotic systems, focusing on the proposals of multimodal, immersive and adaptive interfaces. Section 3 describes the multi-robot missions used to design, develop and validate the interfaces. Section 4 addresses the virtual reality and prediction resources used in the interfaces. Section 5 gives a detailed description of the four interfaces developed by the authors. Section 6 addresses the design of the experiments performed to validate the interfaces. Section 7 discusses the results of the workload and situational awareness tests performed in the experiments. And finally, Section 8 summarizes the main conclusions of the work.

## 2. State of Art

Interfaces are a key element in multi-robot missions, since they manage the interactions between operators and robots. They are influential not only in the discovery and display of information, but also in the generation and transmission of commands. Table 2 compares the interfaces developed in this work with a diverse set of interfaces extracted from recent literature.

This chapter analyzes different types of operator interfaces for multi-robot missions. Section 2.1 addresses multimodal interfaces, Section 2.2 describes immersive interfaces, Section 2.3 is focused on adaptive interfaces and, finally, Section 2.4 collects a set of design guidelines.

### 2.1. Multimodal Interfaces

Multimodal interfaces integrate not only conventional interactions (visual), but also non-conventional ones (aural, tactile...) for the transmission of information and generation of commands. Some of the most common problems of robot operation (limited field of view, degraded depth perception, orientation problems, time delays...) can be addressed by developing multimodal interfaces [20].

On one hand, multimodal displays can improve operator performance by complementing the visual information, or drawing the user’s attention to certain variables. The combination of visual and aural spatial information leads to the enhancement of situational awareness [21]. Haptic interactions can be applied in visual interfaces to draw the operator’s attention to the warnings and their locations [22]. Although the influence of visual, aural and haptic feedbacks on the spatial ability of operator is significant, their effects in teleoperation performance are still unclear [23].

On the other hand, multimodal commands offer even more possibilities than multimodal displays. The idea is to combine voice, touch and gestures to reach simple, natural and fast interactions between operators and robots [24]. There are multiple approaches to command multi-robot missions by means of speech commands [25]. In addition to this, gesture commands can be employed in this context, developing not only hand gestures [26], but also combinations of face poses and hand gestures [27].

All virtual reality interfaces designed for this research (VRI and PVRI) include multimodal displays. Specifically, the sounds of the robots and their environment and the vibration of the controllers are used to support the situational awareness of operators. Since the objective of the study is mission monitoring and not robot commanding, multimodal commands have not been included in the interfaces.

### 2.2. Immersive Interfaces

Immersive interfaces seek to introduce the operator into the mission scenario. For this purpose, they take advantage of multiple technologies (e.g., 3D or 2D cameras, and virtual or augmented reality glasses). The objective is to reproduce the scenario in detail, and improve the situational awareness of the operator. There are two main types of immersive interfaces: augmented reality (AR) and virtual reality (VR).

Augmented reality combines videos streamed by the robots with relevant information about the mission: e.g., maps, terrain elevation, obstacles, robot paths, target locations and other data [28]. Multiple experiments show the potential of AR in the context of multi-robot missions. For instance, an experiment that compared conventional and AR video stream showed that participants find an increased number of targets more accurately with the support of AR [29]. Further works include maps with 3D printouts of the terrain, where multiple live video streams replace robot representations, and with the possibility of drawing the robots’ paths [30].

Virtual reality integrates representations of both real and virtual elements, such as robots, targets, paths and threats, in order to improve the situational awareness of operators. A comparison among multiple displays in the context of multi-UAV missions pointed out that VR glasses could improve the spatial knowledge of operators at the expense of increasing their workload [16]. The aim of the present work is to go further than this study, looking for quantitative and significant conclusions in terms of situational awareness and workload.

Mixed reality is a combination of AR And VR that searches to create new scenarios where users can interact with both real and virtual objects. Although the application of this technology to immersive interfaces looks promising, there is a lack of guidelines for development or cases of use.

For the sake of this research, two virtual reality interfaces (VRI and PVRI) have been designed, developed and validated. These interfaces not only reproduce the scenario, robots and targets, but also display relevant information about the mission.

### 2.3. Adaptive Interfaces

Adaptive interfaces seek to provide agents with information required for making decisions and executing actions; while improving their speed, accuracy, understanding, coordination and workload [31]. For this purpose, they integrate data mining and machine learning algorithms, able to perform operator functions, discover relevant information, and support their decisions.

Several papers found in literature collect guidelines to design intelligent adaptive interfaces (IAIs) [32]. Adaption requires various types of models (knowledge, operators, robots, tasks, world...) to manage information about the missions. Moreover, the adaption process should cover four steps: knowledge acquisition (what is happening?), attention (what adaptation is going to occur?), reasoning (why is it necessary?) and decision making (how is it happening?).

From the interfaces that have been designed for this work, two integrate predictive components: one of the conventional interfaces (PCI) and one of the virtual reality interfaces (PRVI). These components showcase relevant information about the mission extracted from vehicle telemetry, a task usually performed by operators. Specifically, neural networks have been applied to accomplish the information-extracting task, theoretically reducing the operators’ workload.

### 2.4. Design Guidelines

This section attempts to answer the question “How should a good multi-robot interface be?”. For this purpose, a review of interface literature has been carried out and design guidelines have been collected. The main hardware and software requirements obtained from this review are collected in Table 3.

## 3. Multi-Robot Missions

**Figure 1:**
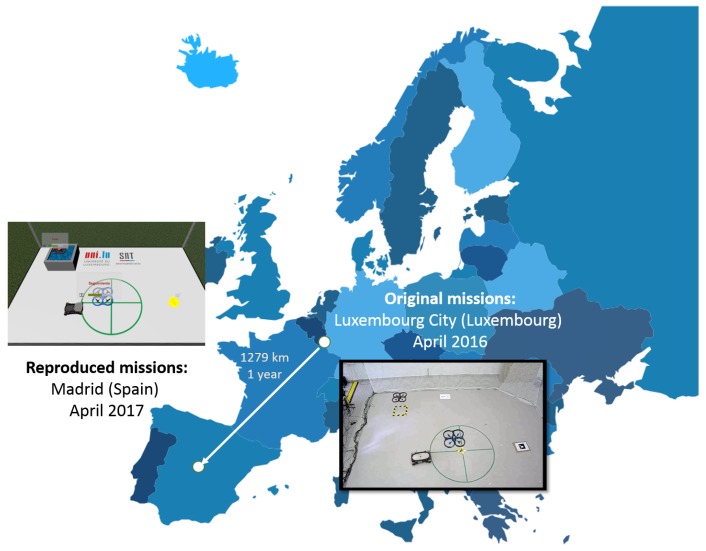
Multi-robot missions performed in Luxembourg in 2016 and virtually reproduced in Madrid in 2017.

A set of multi-robot missions were used in order to evaluate the four interfaces (non-predictive and predictive conventional and non-predictive and predictive virtual reality interfaces). These missions were carried out at the laboratory of the Interdisciplinary Centre for Security, Reliability and Trust (SnT) of the University of Luxembourg in April 2016 [39]. However, as shown in Figure 1, the interface tests took place at the laboratory of Robotics and Cybernetics Group (RobCib) of the Technical University of Madrid (UPM) in April 2017. The missions were reproduced successfully with the aid of stored telemetry and commands, together with video recordings of every session.

The missions’ aim was to control two aerial robots that alternatively performed tasks of detecting-extinguishing fires, and finding-following intruders. The scenario was set up in a 5.35 m × 6.70 m × 5.00 m room with a robot base, a water well and a fire with variable locations. The UAVs had to perform the following tasks to accomplish the mission:

- Begin: The robot switches on and takes-off.
- Surveillance: The robot flies over an area at high altitude with a back and forth pattern to find potential fires.
- Reconnaissance: The robot flies over a list of points at low altitude to check the previously detected fires.
- Capture: The robot flies to the reservoir, descends and loads water.
- Release: The robot flies to the fire, ascends and discharges water over it.
- Go to WP: The robot flies to a waypoint with other purposes: e.g., to leave free the way of the other robot.
- Tracking: The robot follows the suspect across the scenario at low altitude.
- Finish: The robot lands and switches off.

Two *Parrot AR.Drone 2.0* UAVs [40] and a *KUKA Youbot* [41] UGV were employed to perform the missions. The aerial robots were operated through an interface; whereas the ground robot was considered as a target for detection and tracking and, therefore, was out of control for the UAV operator.

A motion capture system *Optitrack* was used to get the accurate position and orientation of aerial and ground robots [42]. This system was able to capture and track the robots around the scenario with a series of infrared cameras together with a set of reflective markers attached to the robots.

All hardware and software components of the missions, including the robots, motion capture system and operator interface, were integrated by means of the Robot Operating System (ROS) open-source framework [43].

The aerial robots rendered a full telemetry record that consisted of state; position and orientation estimations based on visual odometry; angular and linear velocities and accelerations based on IMU readings; battery level; and motor voltage. Additionally, the motion capture system provided accurate readings of the positions and orientations of the aerial and ground robots. Finally, the user interface allowed the operator to send commands with the robot tasks referenced in real time. All this information was stored for future use in ROS bag files, as well as XLSX spreadsheets.

## 4. Resources

This chapter analyzes the main two resources used in the interfaces: the predictive component, which is described in Section 4.1, and the virtual reality, which is detailed in Section 4.2.

### 4.1. Predictive Component

**Figure 2:**
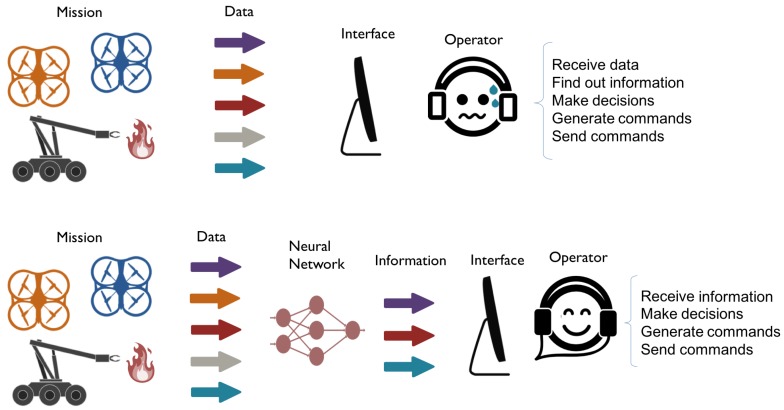
Contribution of prediction to interfaces. (**upper**) The operators of non-predictive interfaces have to receive data, find information, make decisions, generate commands and send them. (**lower**) Predictive interfaces can help the operators performing some functions such as the discovery of information.

As stated above, multi-robot missions pose a series of challenges to human operators, including the management of workload and the preservation of situational awareness. As shown in Figure 2, operators using conventional interfaces must receive data, discover information, make decisions, generate commands and send them. A potential solution to relieve this workload is to transfer functions from the operator to the interface. Specifically, the idea behind this work is to assign the discovery of information to the interface instead of the operator.

An interesting related work can be found in [44], whose goal is to provide robots with human capabilities to perceive, understand and avoid the risks during their missions. For this purpose, the author studies human perception, cognition and reaction against risks and develops a framework to apply these concepts to fleets of aerial robots.

The amount of data generated by a multi-robot mission depends on the number and complexity of robots and tasks. This raw data includes robot states (position, orientation, speed, battery...) and payloads (measurements of sensors, images of cameras, state of actuators...). When the amount of data is exceedingly large, operators may find themselves unable to process everything in order to extract the most relevant parts. Therefore, they might remain oblivious to information such as which task is more critical, which robot requires more attention, which situation has more risks...

A proposed solution to this matter is to automatically determine what information is important from the raw data of mission. For this purpose, all instances of data generated by the mission are considered input variables, and the problem is simplified by selecting three output variables: the task, relevance and risk of each robot at any moment of the mission. Current and next tasks are determined by using Petri nets and decision trees respectively, following methods developed in previous publications [45,46]. Relevance and risk are determined through a manual procedure with four steps: evaluation of human operator, preparation of datasets, neural network training and subsequent validation.

Let’s start with variable definitions:

- Relevance. This variable measures the importance of the robot in a certain situation of the mission. In this work, it is considered as a percentage, which varies from 0% (i.e., the robot is not involved in the mission) to 100% (i.e., it is the unique one that is taking part in the mission). The sum of the relevances of all the robots that take part in the mission must be 100%.
- Risk. This variable measures the potential danger that the robot can suffer in a certain situation of the mission. In this work, it is considered as a percentage, which varies from 0% (i.e., the robot is completely safe) to 100% (i.e., it has suffered an accident). In this case, the risk of one robot is independent of the risks of the rest of the fleet.

**Figure 3:**
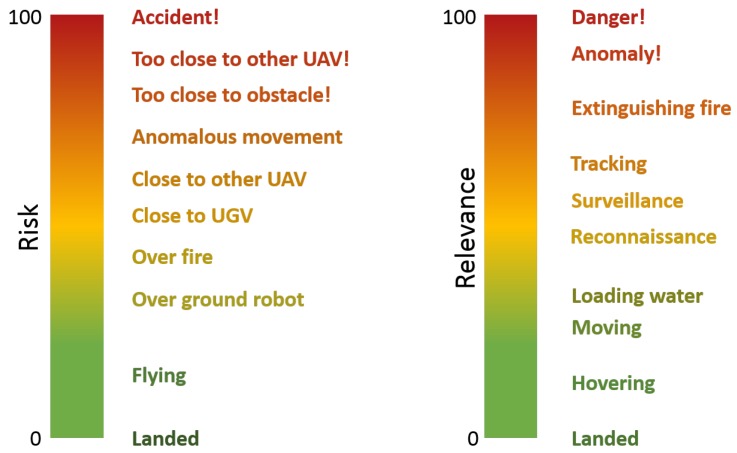
Approximation of evaluation criteria of operator for robot relevance and risk.

The first step of the predictive component development consists of an operator evaluating the relevance and risk of the robots during missions: watching mission videos and noting their values over time. This evaluation was performed with sixteen missions: eight for training and eight for validation. Some of the operator criteria for the evaluation of relevance and risk are shown in Figure 3. On one hand, a robot’s relevance is related to the task that it is performing, together with every possible anomaly and problem. On the other hand, a robot’s risk is influenced by factors such as its proximity to obstacles or other robots. However, it must be remarked that the evaluation is not deterministic, since the operator can provide different outputs for similar inputs.

The second step of the procedure involves dataset preparation. The result is a series of spreadsheet with an average of 590 readings whose columns are the mission variables and whose rows are their values at different timestamps. Mission data includes 101 variables that can be classified as primary variables (60 variables collected by telemetry, such as the position, orientation, battery...), secondary variables (37 variables that are obtained by operating with them, such as the task, distance between robots...), and target variables (relevance and risk of all the robots). These latter variables were subjected to a correlation test: showing absolute inverse correlation between relevances and low correlations between the rest of variables (0.594 between risks, ±0.178 and ±0.395 between relevances and risks).

In third place, neural networks are trained using the operator’s evaluation of risk and relevance. For this purpose, the eight training datasets above mentioned were used, and a back propagation algorithm was implemented in *RapidMiner Studio 7.5*. A total of sixteen NNs have been generated: four NNs (1, 2, 3 and 4 hidden layers) for the four variables (robot 1 and robot 2 relevance and risk).

**Figure 4:**
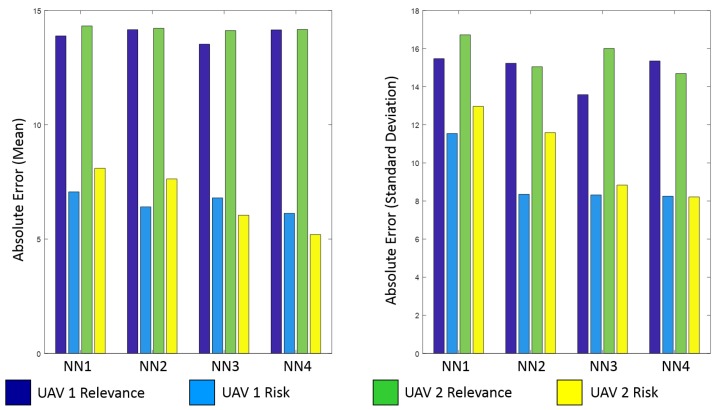
Prediction errors of neural networks.

**Figure 5:**
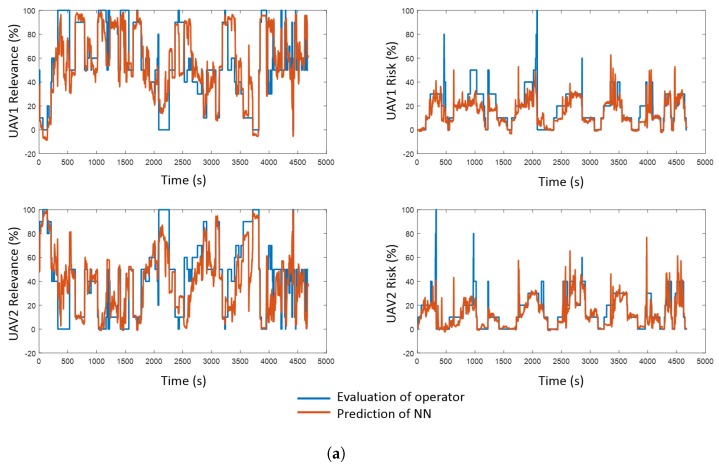

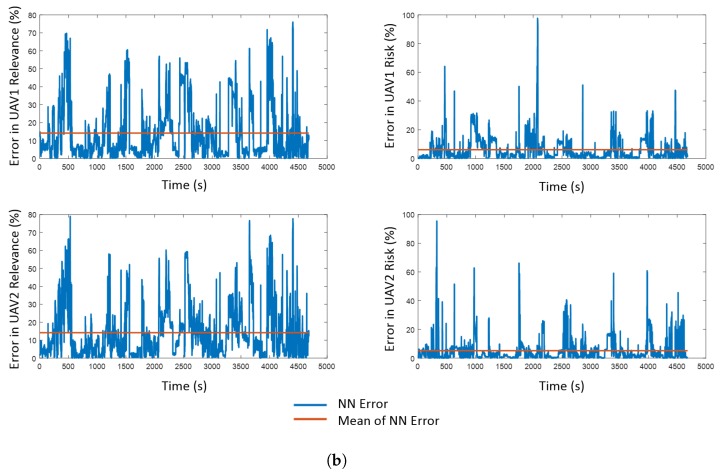
Evaluation of operator vs. prediction of neural networks. (**a**) Direct comparison; (**b**) Absolute error and mean value.

Finally, the fourth step of the procedure involves NN validation with the eight validation datasets. Figure 4 shows the mean and standard deviation of the NN variable prediction errors. It can be appreciated that relevance implies greater errors than risk, probably due to the nature of these variables: the first one oscillates from 0% to 100% with an average of 50%, whereas the second one is usually under 20% and presents some peaks. NNs with four hidden layers have been chosen because they obtain the best results with three of four variables: both risks and the relevance of robot 2. The Figure 5 shows the real and predicted values for the four variables, as well as the absolute error for these variables. As shown, the NNs predict successfully the trends of the variables (i.e., rise and fall), but they are often unable to reach the peaks, explaining the errors above mentioned.

The comparison among the errors of NNs with training and validation datasets reveals a phenomenon of overfitting. Specifically, the errors with training dataset are 5 times lower than with validation one. However, the interfaces do not use directly the values of relevances and risks, but thresholds for these variables. For instance, when the relevance of an UAV is higher than the other UAV, the interfaces select this UAV, or when the risks of an UAV are over 50%, the interfaces show an alert. In this case, the total error of NNs including both false positives and false negatives is 7.64% (11.55% in UAV 1 relevance, 5.1% in UAV 1 risk, 12.28% in UAV 2 relevance and 1.6% in UAV 2 risk). This result is considered as adequate for the correct work of interfaces.

### 4.2. Virtual Reality

The development over the last decade of increasingly immersive systems has provided interface designers with new tools for improving robot operating missions. The range of VR headsets available in the market [47] , shown in Table 4, can be divided in two broad categories: tethered and mobile. Mobile headsets are not able to offer the performance level required for robot operation. They solely consist of lenses that divide a mobile phone screen into two separate images, and current mobile phones cannot offer enough performance level for high end VR applications. Oculus Rift and HTC Vive are the most convenient options for software development, as they are compatible with game engines such as Unity and Unreal Engine 4. This paper addresses an interface designed for HTC Vive using Unity.

**Figure 6:**
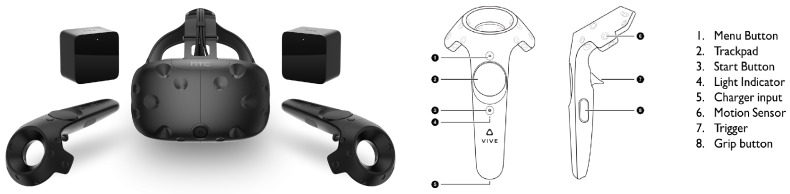
HTC Vive headset and controller.

The HTC Vive virtual reality headset was originally conceived for recreational purposes, but its versatility promotes applications beyond gaming. This system offers both seated and room scale virtual reality. The basic elements of the system are shown in Figure 6. The head-mounted display (HMD) uses one screen per eye, with a refresh rate of 90 Hz and a resolution of 1080 × 1200. An audio jack permits the addition of sound to create a full immersion in the virtual environment. The tracking system involves two light-emitting base stations and photosensors embedded in the HMD and controllers. This system is commonly referred as Lighthouse tracking (where the base stations are the lighthouses), and offers a sub-millimeter precision and a system latency of 22 ms [48]. This last feature is remarkable, as latency is one of the primary factors that cause motion sickness and dizziness when wearing an HMD system [49]. Additional sensors include gyroscopes, accelerometers and a front-facing camera that can be used avoid obstacles within the play area.

The placement of the lighthouses delimits an area of approximately 4.6 m by 4.6 m where the user can move freely, with an accurate tracking of rotation and translation, together with controller movement. Nevertheless, some applications encourage the use of some sort of teleporting system within the VR space. Teleporting can be implemented through the Vive controllers. The HTC Vive set includes two controllers with trackpads, trigger buttons and grip buttons that allow interactions with virtual objects. A basic diagram of the controller elements is shown in Figure 6. These simple and intuitive interactions entail a wide array of potential robot operating commands without the need for any additional devices. Furthermore, the controllers open the door for haptic feedback, as various degrees of vibration can be implemented under certain circumstances.

HTC Vive allows a straightforward interface development through Unity and the Steam VR plugin, which handles all connections between the computer and headset. When modeling a scenario in virtual reality it is important to consider the scale in order to facilitate its navigation. If its size is close to the play area’s size, the scenario can be depicted using a 1:1 scale; but larger scenarios, such as those of outdoor missions, should include options such as bird’s eye view. The possibility of a first person view, attaching the camera to a moving robot, has been discarded, as it most certainly causes virtual reality sickness [50].

One of the advantages of virtual reality, the possibility to reproduce a real scenario, poses an additional challenge when used to portray information within the scene. Non-VR interfaces usually resort to non-diegetic elements, which are not part of the scene per se, but make sense to the operator in the context of their mission. Some examples of non-diegetic elements are mission time, battery percentage, mission state or current command. When creating a VR scene, non-diegetic elements attached to the screen could block the operator’s view and be extremely unpractical. The VR-friendly alternative is the use of diegetic user interface elements. These elements generate an output that can be attached to objects within the game, such as walls (e.g., a sign pointing out the name of an area within the scene), or mobile elements (e.g., battery levels attached to UAVs) [51] .

## 5. Design of Interfaces

This chapter describes with detail the four interfaces that have been developed: non-predictive conventional interface (CI) in Section 5.1, predictive conventional interface (PCI) in Section 5.2, non-predictive virtual reality interface (VRI) in Section 5.3, and predictive virtual reality interface (PVRI) in Section 5.4.

### 5.1. Non-Predictive Conventional Interface

**Figure 7:**
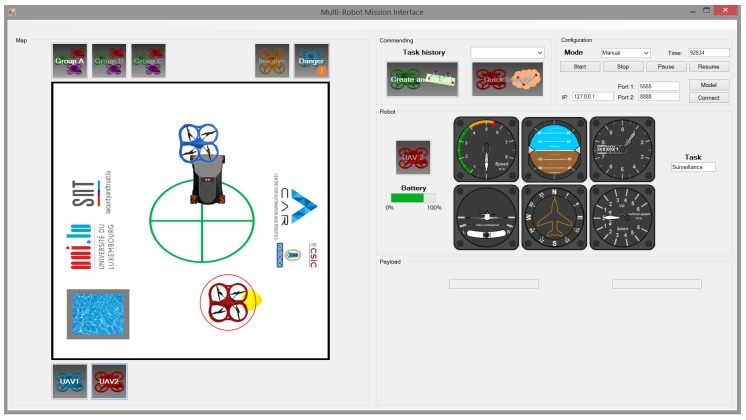
Conventional interface without the predictive component.

This interface is based on the one used to perform the experiments and described in a previous work [39]. This interface is shown in Figure 7 and consists of the following panels (from top-left to down-right): map, commanding, configuration, robot and payload. As the experiments involved mission monitoring but not robot commanding, some panels and some options were not used, mainly the related to commands and payloads.

The map panel shows a scheme of the scenario with its main elements (center, water ...), the location of both UAVs, and the location of fire and UGV when they are discovered. Under the map there is a button for each UAV that the user can click to select it. The robot panel shows the full information of the selected UAV, such as the level of battery, altitude, horizontal and vertical speed and task that is performing. Finally, the configuration panel was used to connect the interface to the telemetry, define the mode (manual in CI and automatic in PCI) and perform the stops and starts.

### 5.2. Predictive Conventional Interface

**Figure 8:**
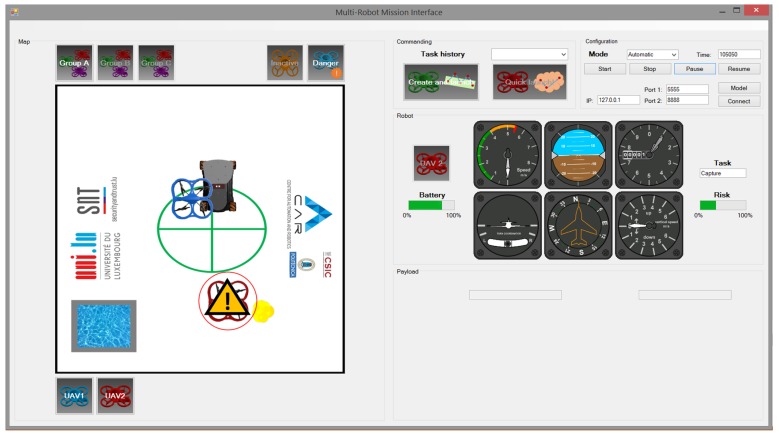
Conventional interface with predictive component.

This interface is similar to the previous one, but it includes predictions to support operators. As mentioned previously, the predictive elements are the relevance and the risk of the UAVs during the mission. The relevance of UAVs was integrated by automatically selecting the most relevant UAV, instead of asking the operator to select it. The risk of UAVs was integrated by means of an indicator in the robot panel, as well as an alert sign in the map when it exceeds the 25% (as shown in Figure 8).

### 5.3. Non-Predictive Virtual Reality Interface

**Figure 9:**
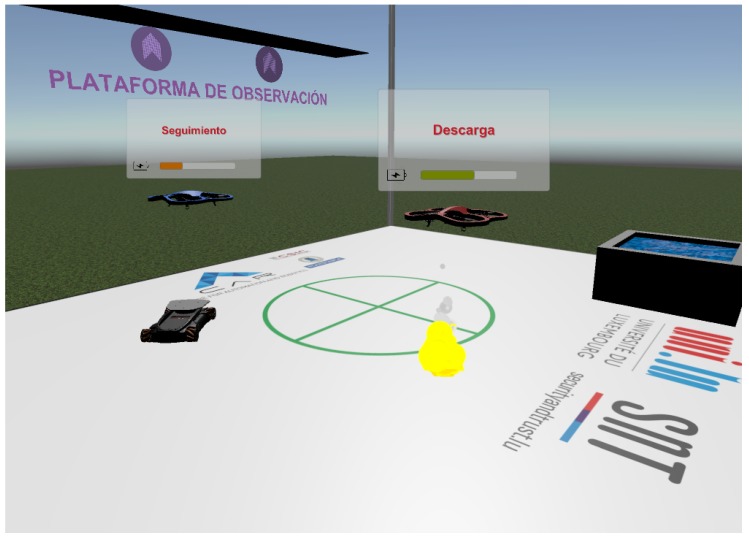
Non-predictive virtual reality interface.

The non-predictive virtual reality interface is based on the Luxembourg experiment scenario, with a full scale representation of all its elements. Figure 9 shows a screen capture of the interface, and will be used to describe its main features. There is a basic scenario with static elements: the floor, with the same design as the conventional interface background—to provide spatial references for the user; the glass walls, that mark out the working area; and the water well, which can also be used as an orientation reference. An observation platform was included to offer the user an additional viewpoint for mission monitoring.

The dynamic interface elements are the two UAVs, the UGV and the fires, together with the HTC Vive controls. Both UAVs are able to fly according to the mission they are performing, with realistic rotor movement and sound. The attached audio source plays in loop a recorded drone hovering sound, which can be used to detect their proximity when they are out of view. The two UAVs are equipped with a translucent screen in which the battery state and name of the task in performance can be checked easily. The battery state bar interpolates its hue between the extreme values of RGB green and red to give an intuitive idea of the battery level. These screens were animated to always face the headset camera, so that the information in them is accesible at all times.

When a mission is played, both the UGV and fire remain hidden until the moment of their detection. Whereas each mission is associated with a fixed fire spawning point, the UGV translates and rotates according to the recorded telemetry data from the real missions. The spawning times for both the UGV and fire have been determined after examining the mission data log.

**Figure 10:**
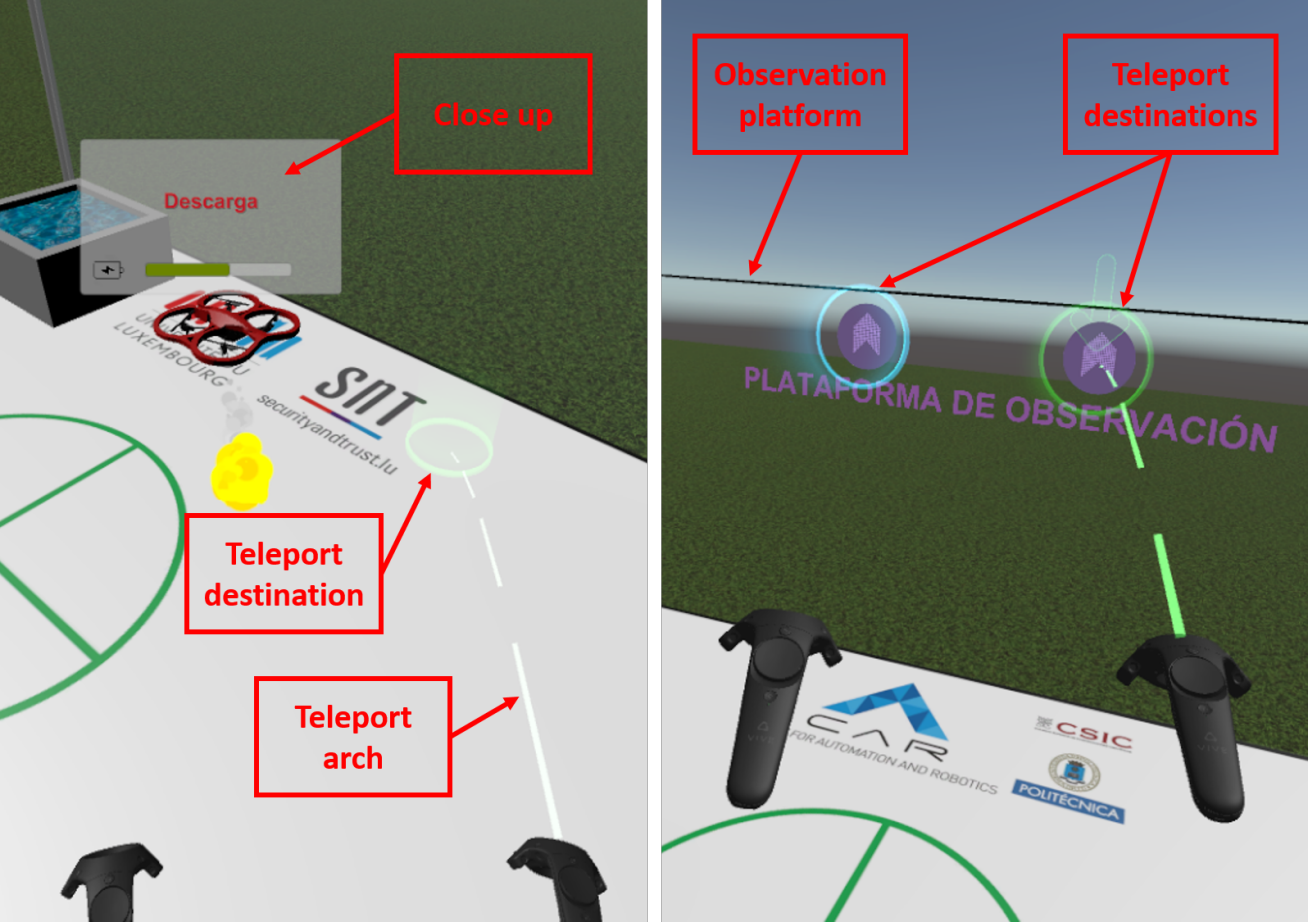
Teleport to floor (**left**) and observation platform (**right**).

Although some walking is allowed with the HTC Vive headset in room scale mode, the interface presents teleporting as the main way to navigate the scene. The teleporting scenario is confined within the working area, delimited by glass walls. When the user presses either of the Vive controller triggers, a parabola comes out of them. Haptic interactions are used to encourage the user to use teleportation. The parabola color, green or red, differentiates between the teleporting and forbidden areas. In order to facilitate access to the observation platform, there are two teleporting points in the wall where it is attached, as can be seen in Figure 10.

### 5.4. Predictive Virtual Reality Interface

**Figure 11:**
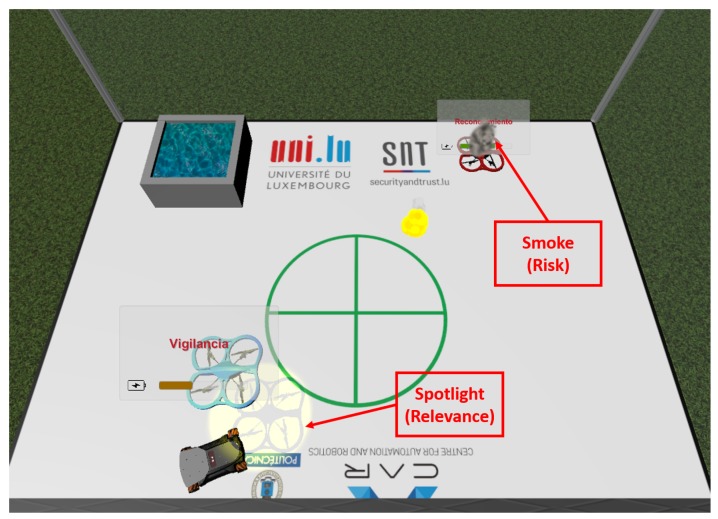
Predictive virtual reality interface.

The predictive virtual reality interface maintains the same elements from the non-predictive version, and adds the predictive component with two new elements that symbolize risk and relevance. There is a spotlight that follows the most relevant drone at every point of the mission, giving hints to the operator about where to look. As for risk, when a risk factor above the threshold is detected, a smoke cloud like the one shown in Figure 11 surrounds the endangered UAV.

## 6. Experiments

**Figure 12:**
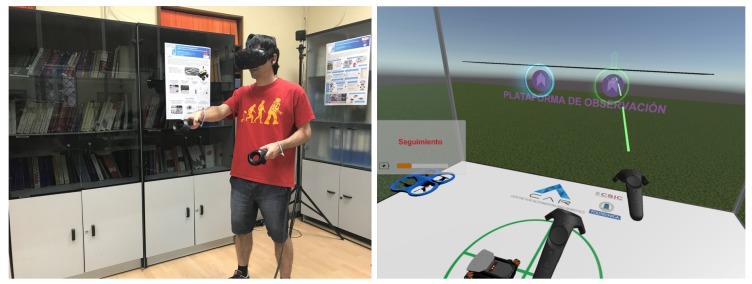
Testing virtual reality interfaces. (**left**) An operator working hard in real world. (**right**) What the operator is doing in virtual world.

A series of experiments were carried out to measure the impact of immersion and prediction in multi-robot mission monitoring. For this purpose, operators used four interfaces integrating the previously mentioned resources to monitor multi-robot missions, as shown in Figure 12.

A total of 24 subjects of different age, genre and expertise were involved in the experiments. Specifically, the participants’ age ranged from 21 to 34 years old, there were 9 women and 15 men and their expertise was classified according to their study level: BSc students (16), MSc students (2), PhD students (5) and PhD (1). The participants were also asked about their experience with videogames and robot missions in a scale from 0 (never) to 5 (work/hobby).

Each participant used two different interfaces to monitor two different missions. The assignation and order of interfaces and missions were planned to compensate the influence of a learning curve on results, as well as to avoid the dependencies between interfaces and missions. In order to explain the design of experiments, the Table 5 shows the interfaces and missions assigned to each participant. This design of experiments provides with 24 samples to compare CIs and VRIs, and 6 samples to compare the pairs CI vs VRI, CI vs PVRI, PCI vs VRI and PCI vs PVRI. In the first case, this figure allows to make conclusions with a statistical confidence level of 95% and a statistical power of 30%. In the second one, the number of samples could be not enough to reach significant conclusions, but the cost of increasing significantly the number of participants or the number of interfaces per participant was not affordable.

Interfaces were evaluated according to the operator workload and situational awareness. We used the NASA-TLX and SAGAT questionnaires in Spanish to obtain values for these variables.

**Figure 13:**
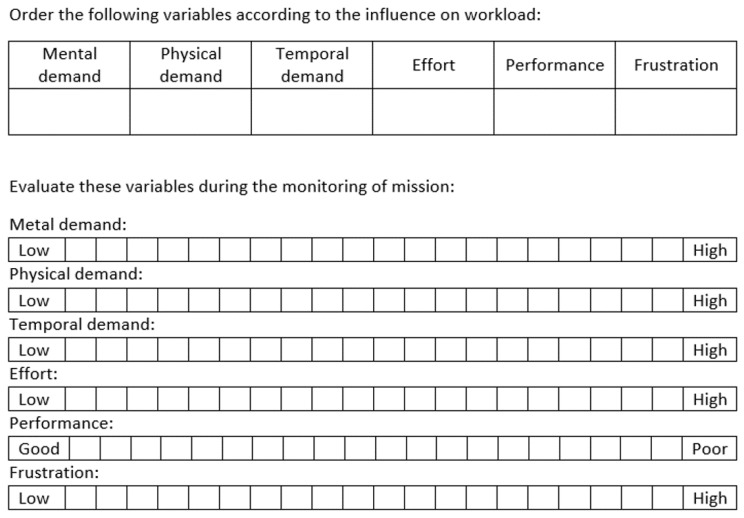
NASA Task Load Index (NASA-TLX): English translation of the questionnaire of the experiments.

The structure of one of the 24 experiments is detailed below:

1. Explanation of missions:
  - The objective of the experiment is to watch multi-robot missions, collect information and answer a series of questions.
  - The goals of the missions are to detect and extinguish fires, and to find and track potential intruders.
  - The mission elements are two drones (one red and another blue), a ground robot, a fire and a water well.
  - The drones perform the following tasks: begin (take-off), surveillance (cover the area to detect fire or intruder), reconnaissance (visit the points to check detections), tracking (follow an intruder), capture (load the water), release (download on fire) and finish (land).
  - It is important to know where the drones are, what tasks they are performing, their battery level, etc.
2. Explanation of interfaces:
  - Conventional interface (CI): The map, the elements (UAVs, fire and UGV), the manual selection of UAV and the information (battery and task).
  - Predictive conventional interface (PCI): The map, the elements (UAVs, fire and UGV), the predictive components (spotlight and alert), the autonomous selection of UAV and the information (battery and task).
  - Virtual reality interface (VRI): The environment (scenario and platform), the teleport mechanism, the elements (UAVs, fire and UGV) and the information (battery and task).
  - Predictive virtual reality interface (PVRI): The environment (scenario and platform), the teleport mechanism, the elements (UAVs, fire and UGV), the predictive components (spotlight and smoke) and the information (battery and task).
3. Annotation of user information: Age, genre and expertise.
4. NASA-TLX (weighing): The user puts into order six variables (mental, physical and temporal demands, effort, performance and frustration) according to their estimated influence on workload (as seen in Figure 13).
5. Test of interface #1:
  - Start: The user starts to monitor the multi-robot mission.
  - Stop #1: We notify the user and, after ten seconds, stop the interface.
  - SAGAT (first part): The user answers some questions about the past, current and future locations and states of UAVs. The questionnaire is explained in further detail below.
  - Resume: The user starts again to monitor the multi-robot mission.
  - SAGAT (second part):The user answers some questions about the past, current and future locations and states of UAVs. The questionnaire is explained in further detail below.
6. Test of interface #2: The same procedure applied in interface #1.
7. NASA-TLX (scoring): The user evaluates both interfaces according to six variables (mental, physical and temporal demands, effort, performance and frustration) and marks values from 0 to 20 (as shown in Figure 13).
8. Annotation of user observations.

**Figure 14:**
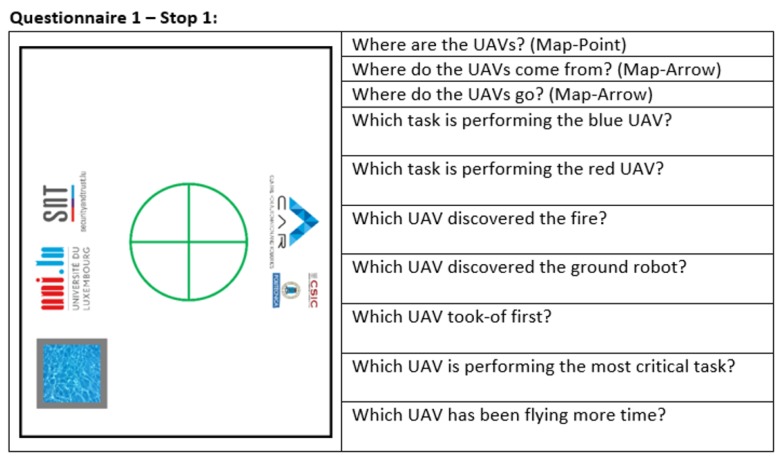
Situation awareness global assessment technique (SAGAT): English translation of the questionnaire of the experiments.

As mentioned in the experiment layout, missions were stopped twice to pass the SAGAT questionnaire. The first stops were after 1 to 2 min of mission, whereas the second ones were after 2 or 3 min. Along each one of these stops, the participants had to answer 10 questions. Five of these questions were fixed: the locations of UAVs, their past and future evolution and the tasks they were performing. The rest of the questions depended on the mission and included the perceived distance from UAVs to fire, water or UGV, battery levels of UAVs, which UAV discovered fire or UGV, etc. An example is shown in Figure 14.

## 7. Results

This chapter presents the results of the previously stated experiments. Table 6 summarizes the average workload and situational awareness scores per interface obtained from the NASA-TLX and SAGAT questionnaires respectively. Additionally, this table shows the positive and negative user reviews. A quick analysis of the means show that immersive interfaces are better than their conventional counterparts in terms of workload and situational awareness, whereas the effects of the predictive components depend on the interface (conventional or virtual reality) and the variable (workload or situational awareness). These results and their statistical relevance are discussed in the following sections.

### 7.1. Workload

As shown in Table 6, the interfaces can be arranged by means of increasing workload as follows: VRI, PVRI, CI and PCI. This order points out that virtual reality interfaces tend to reduce operator workload, whereas the predictive components tend to increase it. Nevertheless, these results could be influenced by the procedure and not be statistically significant.

In order to check the statistical significance, the results have been split according to the workload variables (mental, physical and temporal demand, effort, performance, and frustration) and the interfaces (on one hand, CI vs. PCI vs. VRI vs. PVRI, and, on the other hand, CI and PCI vs. VRI and PVRI).

**Figure 15:**
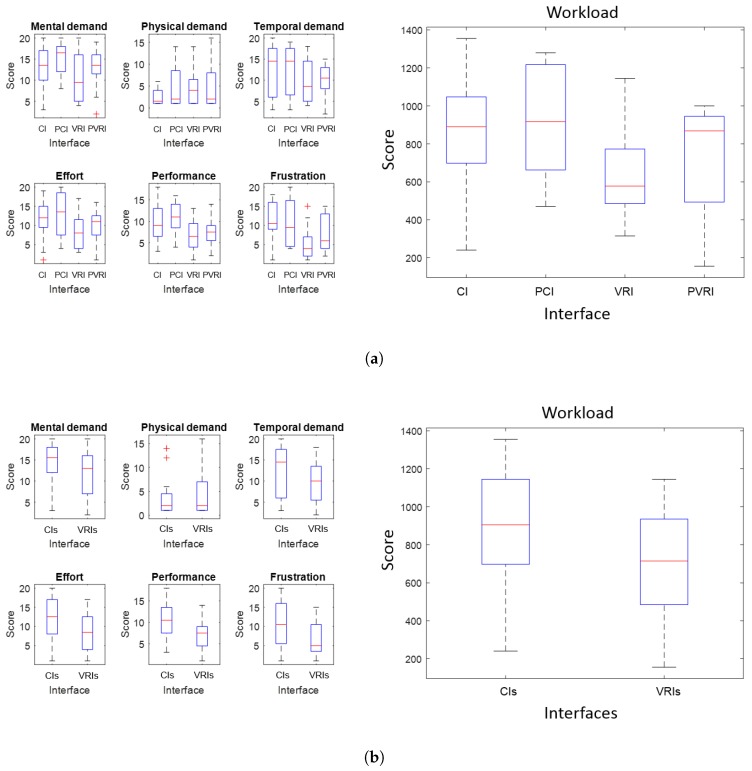
Box and whiskers diagrams for the workload and its variables. (**a**) Plot with the four interfaces: Conventional (CI), Predictive Conventional (PCI), Virtual Reality (VRI) and Predictive Virtual Reality (PVRI). (**b**) Plot with two groups of interfaces: Conventional (CIs) and Virtual Reality (VRIs).

Figure 15a shows box and whisker diagrams of the workload and its variables for the four interfaces. The one-way analysis of variance (ANOVA) of the complete dataset shows there are no significant differences between the workload of the four interfaces (F = 2.26, *p* = 0.1061). Similar studies for the variables of workload show there are significant differences with α = 0.05 in performance (F = 2.59, *p* = 0.065) and frustration (F = 3.37, *p* = 0.0266), while these differences do not apply to the remaining variables.

Figure 15b shows the same diagrams for the interfaces now grouped in two blocks: conventional and virtual reality. The one-way ANOVA of this dataset shows there are significant difference with α = 0.05 between the workload of both groups of interfaces (F = 5.58, *p* = 0.0225). Similar studies for workload variables keep these significant differences in effort (F = 4.54, *p* = 0.0384), performance (F = 7.56, *p* = 0.0085) and frustration (F = 8.47, *p* = 0.0055). Once again, these differences do not apply to the other three variables.

Finally, the *t*-test for each pair of interfaces provides the results of Table 7. It can be appreciated that, considering α = 0.05, VRI is significantly better than CI and PCI in terms of workload. The rest of the differences between the pairs of interfaces cannot be considered as significant.

To sum up, we can assert that workload is significantly lower in virtual reality interfaces compared to conventional ones, both predictive and non-predictive. The effects of prediction on workload seem to be negative, but are nevertheless non significant. These unexpected results were probably due to the operators’ need of training to interpret properly the predictive cues of the interfaces.

### 7.2. Situational Awareness

As fas as situational awareness is concerned, Table 6 shows that the interfaces can be arranged in the following order: PVRI, VRI, CI and PCI. This order points out the virtual reality interfaces tend to increase the situational awareness of operators, whereas the effects of the predictive components depend on the interface. However, the statistical significance of these results must be checked.

**Figure 16:**
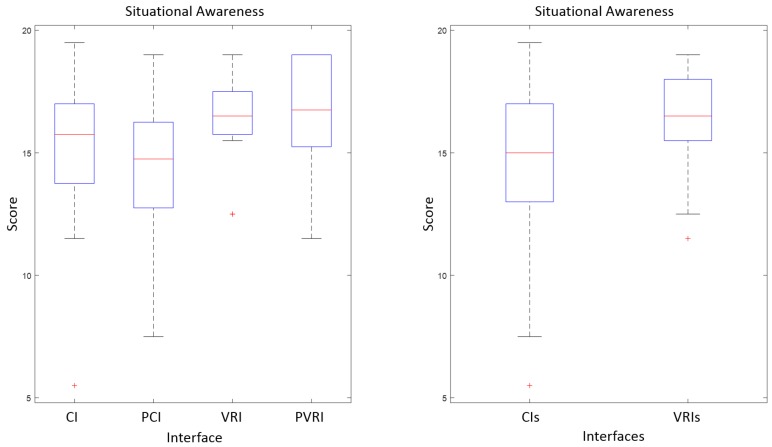
Box and whisker diagrams for the situational awareness with four interfaces (**left**) and two groups of interfaces (**right**).

Figure 16 shows box and whisker diagrams for situational awareness of the four interfaces (CI, PCI, VRI and PVRI) and the two groups of interfaces (CIs and VRIs). The one-way ANOVA with α = 0.05 of the four interfaces does not provide significant results (F = 1.49, *p* = 0.2296). However, the same analysis with the two groups of interfaces detects a significant difference (F = 4.37, *p* = 0.042).

Finally, the t-test of each pair of interfaces provides the results of Table 8. In this case, the differences between pairs of interfaces are not statistically relevant. However, the differences of VRI and PVRI over PCI are closer to α = 0.05 than the rest of them.

To sum up, we can state that virtual reality significantly improves the situational awareness of operators, since their score in SAGAT questionnaire is significantly higher than the score of conventional interfaces. In this case, the effects on prediction on situational awareness depend on the interface, probably due to the fact that prediction implementation with virtual reality is easier to understand than the implementation in a conventional interface. Nevertheless, these effects once again are not significant.

### 7.3. User Evaluation

Finally, the questionnaires had a field of observations where users could write comments and suggestions. A total of 39 reviews about the interfaces have been collected: 15 were positive and 24 were negative. The results can be seen in Table 6 and are described below.

The conventional interface had 7 negative reviews, mainly related to the complexity of understanding the information of the mission (3) and the need to select the UAVs to get their full information (2).

The predictive conventional interface also received 7 negative reviews, in this case about the amount of information (3) and the complexity to understand some variables (3).

The virtual reality interface received 5 positive and 5 negative reviews. The positive comments pointed out that the interface is easy to understand (3) and fun to use (2), whereas the negative ones reported different problems about the observation platform (3), including perspective difficulties and physical discomfort.

The predictive virtual reality interface received 10 positive and 5 negative reviews. In this case, the positive comments stated the interface is easy to understand (3) and comfortable (3), they also praised the usefulness of teleporting (1) and prediction (1). On the other hand, the negative ones reported difficulties to read some variables (3) and the mentioned problems with the observation platform (2).

By way of curiosity, the situational awareness (SA) and workload (W) scores do not show correlation with the experience with videogames (V) and robot missions (RM). Specifically, these are the correlation coefficients: V-SA (0.0102), RM-SA (−0.2048), V-W (0.0855) and RM-W (0.0675).

## 8. Conclusions

The scenarios with multiple robots and single operator suppose a challenge in terms of human factors. In these scenarios, the operator workload can be excessive, since they have to receive data, discover information, make decisions and send commands. Their situational awareness may also decline at certain moments of the mission, which can lead to errors in perception and decision-making that can cause accidents. This work analyzes the impact of immersive and predictive interfaces on these human factor problems.

For this purpose, four interfaces have been developed: conventional (CI), predictive conventional (PCI), virtual reality (VRI) and predictive virtual reality (PVRI). These interfaces include multimodal interactions (VRI and PVRI), immersive technologies (VRI and PVRI) and predictive components (PCI and PVRI).

Twenty-four operators have monitored eight multi-robot missions using the four interfaces and answered NASA-TLX and SAGAT questionnaires. The results of these tests showed that virtual reality interfaces significantly improve the situational awareness and reduce the workload (specifically, the components related to effort, performance and frustration). The effects of predictive components depend on the interface (negative in PC and positive in PVRI) and are not statistically significant.

Future works should continue this line and address diverse topics, such as the development and integration of multimodal commands, and the implementation of predictive components in virtual reality interfaces.

## Figures and Tables

**Table 1:** Human factors in multi-robot missions.

Issue	Problems	Detection	Solution
Workload	Excessive: Inefficiency	Physiological signals	Adjust autonomy
	and errors	Test (NASA-TLX)	Transfer functions
Situational	Lack: Inefficiency	Actions and performance	Immersive interface
awareness	and errors	Test (SAGAT)	Filter information
Stress	Boredom: Human errors	Physiological signals	Adjust autonomy
	Anxiety: Human errors	Test (NASA-TLX)	Filter information
Trust	Mistrust: Human errors	Reactions	Adjust autonomy
	Overtrust: Machine errors	Survey	Train operators

**Table 2:** The interfaces of this work, against a diverse set from literature.

Reference	Robots	Operators	Multimodal	Immersive	VR	AR	Adaptive
[10]	1	1	Yes	No	No	No	No
[11]	40	1	Yes	No	No	No	No
[12]	50–200	1	No	No	No	No	No
[13]	4	1	No	No	No	No	No
[14]	3	1	No	No	No	No	No
[15]	4	1	No	No	No	No	No
[16]	3	1	No	Yes	Yes	No	No
[17]	10	1	No	Yes	No	No	No
[18]	1	1	No	Yes	No	Yes	No
[19]	N	1	No	No	No	No	Yes
CI	2	1	No	No	No	No	No
PCI	2	1	No	No	No	No	Yes
VRI	2	1	Yes	Yes	Yes	No	No
PVRI	2	1	Yes	Yes	Yes	No	Yes

**Table 3:** The requirements for interfaces collected by literature.

Reference	Requirement
[33]	Resistance to weather, environment and harsh conditions.
[34]	Reduction of the amount of information.
[35]	Adaptation to the preferences of operator.
[36]	Guidance of operator attention to relevant information.
[37]	Integration of robot position, health, status and measurements in the same displays.
[38]	Use of maps to show information about robots and mission.

**Table 4:** Study of virtual reality (VR) headsets.

Name	Type	Hardware Required
Sony PlayStation VR	Tethered	PlayStation 4
HTC Vive	Tethered	PC
Oculus Rift	Tethered	PC
Google Daydream View	Mobile	Daydream compatible phone
Samsung Gear VR	Mobile	Latest Samsung Galaxy models
Homido VR	Mobile	Android and iOS phones
FreeFly VR	Mobile	Android and iOS phones
Google Cardboard	Mobile	Android and iOS phones

**Table 5:** Design of experiments.

Subject	Interface	Mission
O1	VRI and CI	M1 and M2
O2	VRI and PCI	M3 and M4
O3	PVRI and CI	M5 and M6
O4	PVRI and PCI	M7 and M8
O5	CI and VRI	M8 and M7
O6	CI and PVRI	M6 and M5
O7	PCI and VRI	M4 and M3
O8	PCI and PVRI	M2 and M1
O9	VRI and CI	M8 and M7
O10	VRI and PCI	M6 and M5
O11	PVRI and CI	M4 and M3
O12	PVRI and PCI	M2 and M1
O13	CI and VRI	M1 and M2
O14	CI and PVRI	M3 and M4
O15	PCI and VRI	M5 and M6
O16	PCI and PVRI	M7 and M8
O17	VRI and CI	M5 and M6
O18	VRI and PCI	M7 and M8
O19	PVRI and CI	M8 and M7
O20	PVRI and PCI	M6 and M5
O21	CI and VRI	M4 and M3
O22	CI and PVRI	M2 and M1
O23	PCI and VRI	M1 and M2
O24	PCI and PVRI	M3 and M4

**Table 6:** Summary of the results of the experiments.

Interface	Workload (NASA-TLX)	Situational Awareness (SAGAT)	Evaluation (+/−)
CI	853	14.91	0/7
PCI	915	14.33	0/7
VRI	638	16.25	5/5
PVRI	740	16.46	10/5

**Table 7:** T-test with pairs of interfaces in terms of NASA-TLX scores.

	VRI	PVRI
CI	CI > VRI	CI > PVRI
	Significant (*p* = 0.0180)	Non-significant (*p* = 0.3716)
PCI	PCI > VRI	PCI > PVRI
	Significant (*p* = 0.0237)	Non-significant (*p* = 0.1008)

**Table 8:** T-test with pairs of interfaces in terms of SAGAT scores.

	VRI	PVRI
CI	CI < VRI	CI < PVRI
	Non-significant (*p* = 0.2584)	Non-significant (*p* = 0.3461)
PCI	PCI < VRI	PCI < PVRI
	Non-significant (*p* = 0.1011)	Non-significant (*p* = 0.0978)

## Supplementary Materials

A video of the work can be found in https://www.dropbox.com/sh/4cdtpd2vn27mgav/AABwBlwfI8bzZte3psfwSi5Fa?dl=0.

## Abbreviations

The following abbreviations are used in this manuscript:

ANOVA	Analysis of Variance
AR	Augmented Reality
IAI	Intelligent Adaptive Interface
NN	Neural Network
UAV	Unmanned Aerial Vehicle
UGV	Unmanned Ground Vehicle
VR	Virtual Reality
CI	Conventional Interface (developed by the authors)
PCI	Predictive Conventional Interface (developed by the authors)
PVRI	Predictive Virtual Reality Interface (developed by the authors)
VRI	Virtual Reality Interface (developed by the authors)
